# Supplementary material for: Differences in the Profile of Aromatic Metabolites in the Corresponding Blood Serum and Cerebrospinal Fluid Samples of Patients with Secondary Bacterial Meningitis
Source: Metabolites. 2025 Aug 3;15(8):527. doi: 10.3390/metabo15080527 (PMC12388189; doi:10.3390/metabo15080527)
Supplement: Supplementary file 1 [file metabolites-15-00527-s001.zip › metabolites-3668743 Suppl S1.pdf]

Differences in the profile of aromatic metabolites in the corre-sponding blood serum and cerebrospinal fluid samples of pa-tients with secondary bacterial meningitis

Alisa K. Pautova, Peter A. Meinarovich, Vladislav E. Zakharchenko, Pavel D. Sobolev, Natalia A. Burnakova, Natalia V. Beloborodova

Supplementary S1. Results of blood and cerebrospinal fluid clinical and biochemical analyses and concentrations of aromatic metabolites in the blood serum and cerebrospinal fluid of patients with long-term sequelae of severe brain damage.

Sample	**1**	**2**	**3**	**4**	**5**	**6**	**7**	**8**
**class (meningitis)**	0	0	0	0	0	0	0	0
**Hemoglobin**	95	110	85	113	134	86	139	117
**Hematocrit**	29.7	33.4	26.5	34.9	38.6	26.6	41.3	35.1
**Platelets**	451	298	367	277	205	325	228	169
**Total protein**	49.5	69.5	67.9	53.7	57.9	54.7	69.8	57.6
**Glucose**	3.32	5.61	5.82	10	5.82	6.22	5.86	6.17
**Albumin**	25.9	40.9	33.8	22	32.4	33.88	40.9	34.7
**Creatinine**	39.8	40.7	76.8	79.9	46.7	101.2	86.3	60.6
**Urea**	4.4	3.1	8.8	5.3	2.5	7.2	4.2	5
**C-reactive protein**	25.93	1.7	55.79	265.58	90.61	80.81	0.6	0.35
**INR**	1.08	1.04	1.32	1.33	1.208	1.08	1.24	1.15
**APTT**	27.6	24.7	36.2	26.8	31.74	27	25.2	25.2
**Cytosis csf**	4	1	16	71	1	19	8	6
**Neutrophiles csf**	27	45	72	71	66	68	48.4	42
**Glucose csf**	2.2	4	4	10.3	3.8	3	5.6	5
**Protein csf**	1.1	0.5	0.6	4.8	0.3	0.8	0.5	0.5
**Lymphocytes csf**	72	100	27	26	100	32	100	56
**p-HPhLA_s**	839.6	431.2	990.4	4309	765.2	1393	1694	855.1
**p-HBA_s**	18700	17690	5829	73220	8278	11380	50320	36120
**p-HPhAA_s**	484.5	1535	1018	5678	0	749.7	569.6	2377
**PhPA_s**	0	0	0	0	0	0	0	0
**p-HPhPA_s**	42.1	0	16.26	55.07	0	23.59	67.76	39.38
**PhLA_s**	1231	939.6	1785	13740	1815	3391	2986	1555
**5HIAA_s**	40.93	66.34	105.2	0	40.79	0	0	40.15
**3ILA_s**	425.7	435.4	614.5	1001	0	626.7	496.9	357.4
**3ICA_s**	25.96	34.36	42.94	59.43	16.46	40.04	60.33	27.5
**3IAA_s**	234.2	2891	1118	881.8	270.5	716.7	1145	840.4
**3IPA_s**	0	0	0	0	0	0	823.3	422.7
**p-HPhLA_csf**	439	369.5	335.2	386.3	123.6	444.5	1238	1047
**p-HBA_csf**	36.94	56.78	26.46	79.38	9.11	35.92	60.51	51.46
**p-HPhAA_csf**	70.27	131.2	100.9	372.9	15.27	52.17	222.6	1075
**PhPA_csf**	0	0	0	0	0	0	0	0
**p-HPhPA_csf**	0	0	0	0	0	0	11.29	8.822
**PhLA_csf**	35.86	42.82	34.07	278.1	13.22	76.01	67.67	54.2
**5HIAA_csf**	118.1	22.1	0	0	30.65	0	377	322.3
**3ILA_csf**	8.569	5.04	0	36.58	0	0	15.2	13.78
**3ICA_csf**	7.621	13.18	11.44	39.4	4.431	13.12	11.63	7.818
**3IAA_csf**	18.47	24.47	21.53	34.22	13.14	0	163.5	133.7
**3IPA_csf**	6.11	0	0	5.317	0	2.04	10.27	6.99

Sample	9	10	11	12	13	14	15	16
**class (meningitis)**	0	1	1	1	1	1	0	0
**Hemoglobin**	80	77	119	82	77	90	122	129
**Hematocrit**	25	22.8	33.5	25.1	24.1	28.8	35.3	38.5
**Platelets**	724	148	28	36	155	147	327	390
**Total protein**	51.9	50.4	50.4	66.5	51.6	52.2	60	68.5
**Glucose**	6.06	10.1	10.1	7.56	8.1	11.62	5.77	4.87
**Albumin**	32.92	28.7	28.7	25.9	25.5	22.3	36.8	40.9
**Creatinine**	33.1	46.3	46.3	62.3	66.4	58.7	58	59.4
**Urea**	2.9	6.5	6.5	12.1	9.8	9.7	3.1	3.1
**C-reactive protein**	71.15	248.01	248.01	51.36	31.6	45.78	2.57	0.64
**INR**	1.22	1.08	1.08	1.16	1.13	1.01	1.13	1.77
**APTT**	33.44	31.1	31.1	18.9	19.8	25.8	28.6	29.6
**Cytosis csf**	2	11	11	286	2833	2085	61	16
**Neutrophiles csf**	31	50	50	91	96	93	54	20
**Glucose csf**	3.8	3.7	3.7	0.1	0.5	0.4	1.7	1.6
**Protein csf**	0.2	0.5	0.5	5.9	6.7	7.8	0.9	1.1
**Lymphocytes csf**	67	48	48	5	2	5	43	78
**p-HPhLA_s**	960.1	3231	4260	1378	2330	2184	695.8	584.9
**p-HBA_s**	7689	16380	19800	29750	10300	19520	10440	12660
**p-HPhAA_s**	1791	299.4	260	2745	7211	4877	1531	2299
**PhPA_s**	299	0	0	0	0	0	0	0
**p-HPhPA_s**	111.3	0	0	0	9.816	12.2	31.46	12.38
**PhLA_s**	5243	12300	13510	4610	8459	8132	2782	13840
**5HIAA_s**	47.93	61.69	70.59	35.59	93.76	35.87	40.28	35.27
**3ILA_s**	326.8	1600	957.2	829.6	1742	1205	765.9	796.2
**3ICA_s**	19.99	41.74	43.71	30.22	24.74	50.66	31.77	32.54
**3IAA_s**	543.6	362.3	0	0	642.4	497.8	974.9	721.7
**3IPA_s**	0	0	0	0	0	0	336.1	0
**p-HPhLA_csf**	1063	4111	5089	8195	4206	2947	684.2	540.8
**p-HBA_csf**	27.23	550.1	661.5	98.75	11.1	19.18	16.76	29.91
**p-HPhAA_csf**	150.5	741.9	616.1	2470	5607	3167	268.6	314.6
**PhPA_csf**	0	0	0	0	0	0	0	0
**p-HPhPA_csf**	8.225	0	0	0	0	8.534	0	0
**PhLA_csf**	60.26	370.1	430.8	6953	848.5	622.2	71.95	108.3
**5HIAA_csf**	281.5	118.6	126.4	259.2	376.5	381.5	78.11	0
**3ILA_csf**	4.737	15.05	14.8	1959	558.3	475.7	19.91	17.23
**3ICA_csf**	4.83	14.29	30.98	30.01	19.55	13.11	5.199	7.635
**3IAA_csf**	32.97	29.99	35.81	378.3	217.2	247.1	43.17	23.8
**3IPA_csf**	0	0	0	4.103	9.936	6.589	3.957	0

Sample	**17**	**18**	**19**	**20**	**21**	**22**	**23**	**24**
**class (meningitis)**	0	1	1	1	1	1	1	1
**Hemoglobin**	125	86	115	111	81	73	104	102
**Hematocrit**	37.2	26.3	33.9	32.7	24.1	23.1	31.5	31.7
**Platelets**	285	612	272	194	630	385	208	276
**Total protein**	64.5	64.4	55	51.1	49	47	52.1	50.6
**Glucose**	4.06	3.98	5.44	7.62	6.53	16.23	6.18	4.89
**Albumin**	41.2	30.44	31.18	32	24.3	26.9	35.9	31.2
**Creatinine**	88.5	42.1	99.7	49.8	53.5	77.3	53.9	66.6
**Urea**	7.5	2.2	5.2	6.4	2.1	4.1	18	20.5
**C-reactive protein**	16.17	70	66.46	30.6	7.99	26.57	11.85	21.23
**INR**	0.97	1.94	1.63	0.97	1.09	1.15	0.99	1.12
**APTT**	26.2	71.6	55.7	45.1	37.9	46.4	42	44.5
**Cytosis csf**	7	37500	244	10000	1830	1586	147	297
**Neutrophiles csf**	5	94	92	83	88	88	88	92
**Glucose csf**	1.8	0.1	4.4	1.5	0.7	31.9	1.4	0.7
**Protein csf**	1.1	7.2	3.8	1	0.8	1.7	1.1	1.7
**Lymphocytes csf**	94	3	6	7	10	9	8	5
**p-HPhLA_s**	884.7	1291	1676	720.4	1040	2077	1509	1665
**p-HBA_s**	8067	35480	15310	8473	7437	7523	6489	7791
**p-HPhAA_s**	3993	499.7	2636	771.6	254.4	637	8552	5512
**PhPA_s**	0	0	0	0	0	0	0	0
**p-HPhPA_s**	14.71	0	0	0	0	0	18.25	0
**PhLA_s**	1670	3431	4774	5602	1991	3894	5971	5949
**5HIAA_s**	77.69	70.52	102.2	65.71	47.99	94.66	52.38	79.95
**3ILA_s**	997	605.7	932.8	1996	1149	1547	2923	2178
**3ICA_s**	32.89	38.61	25.69	25.04	32.11	31.06	26.39	43.23
**3IAA_s**	1318	0	0	335.6	313.3	911.4	969.7	623.5
**3IPA_s**	0	0	0	0	0	0	375.1	0
**p-HPhLA_csf**	263.8	2416	1903	1831	2578	3830	1734	2491
**p-HBA_csf**	22.71	24.01	18.66	42.09	44.61	149.7	42.08	35.05
**p-HPhAA_csf**	387.1	361.9	1827	341.9	161	331.8	4089	4413
**PhPA_csf**	0	0	0	0	0	0	0	0
**p-HPhPA_csf**	0	0	0	0	0	0	0	0
**PhLA_csf**	29.49	735.5	428.6	571.6	527	678	421.4	668.4
**5HIAA_csf**	0	69.86	79.83	72.35	61.56	123.6	31.65	326.9
**3ILA_csf**	11.26	471.7	155.7	103.4	63.73	84.42	58.37	202.5
**3ICA_csf**	12.9	12.46	9.386	13.41	29.16	15.83	10.57	10.27
**3IAA_csf**	73.64	83.76	95.5	36.86	22.85	89.99	49.79	120.5
**3IPA_csf**	0	0	0	3.723	0	0	3.534	4.269

Sample	**25**	**26**	**27**	**28**	**29**
**class (meningitis)**	1	0	0	0	0
**Hemoglobin**	94	130	125	136	102
**Hematocrit**	28.8	38.1	35.9	40	30
**Platelets**	116	236	262	266	288
**Total protein**	36.1	62	63.3	64.8	55
**Glucose**	5.44	5.64	5.55	7.57	7.09
**Albumin**	25	37.3	37.7	37.1	30
**Creatinine**	52.5	69.8	74.9	82.4	80.1
**Urea**	15.9	3.9	2.9	4.7	4.9
**C-reactive protein**	15.39	0.7	0.7	61.71	82.39
**INR**	1.15	1.12	1.24	1.33	1.17
**APTT**	43.3	26.4	31.3	29.3	26.7
**Cytosis csf**	2133	24	39	4	16
**Neutrophiles csf**	96	22	16	55	61
**Glucose csf**	1.3	2.6	3.1	4	3.6
**Protein csf**	4.5	2.6	0.9	0.9	0.5
**Lymphocytes csf**	2	74	81	41	30
**p-HPhLA_s**	2165	1232	1187	1516	1415
**p-HBA_s**	6360	23170	21030	8452	9698
**p-HPhAA_s**	5854	409.1	461.5	374.4	1458
**PhPA_s**	0	0	0	0	0
**p-HPhPA_s**	14.25	146.6	86.8	33.22	43.96
**PhLA_s**	4842	2108	1727	3751	4170
**5HIAA_s**	111.2	37.94	60.79	0	0
**3ILA_s**	2103	642.7	574.9	500.4	645.4
**3ICA_s**	19.25	33.55	38.97	30.15	33.18
**3IAA_s**	509.1	1922	1131	492.4	1208
**3IPA_s**	204.5	587.4	215.1	0	0
**p-HPhLA_csf**	2410	390.1	649.7	308.8	114.2
**p-HBA_csf**	47.82	34.29	165.4	7.553	27.87
**p-HPhAA_csf**	3894	105.4	191.2	33.1	26.52
**PhPA_csf**	0	0	0	0	0
**p-HPhPA_csf**	0	8.412	0	0	0
**PhLA_csf**	537.5	48.94	48.23	27.86	52.06
**5HIAA_csf**	143.1	41.53	42.13	221.8	0
**3ILA_csf**	482.9	9.313	7.549	4.475	0
**3ICA_csf**	11.72	10.49	21.56	5.819	15.53
**3IAA_csf**	148.1	142.6	31.48	23.78	11.55
**3IPA_csf**	23.27	22.85	4.003	6.604	0
