# Supplementary material for: Differences in the Profile of Aromatic Metabolites in the Corresponding Blood Serum and Cerebrospinal Fluid Samples of Patients with Secondary Bacterial Meningitis
Source: Metabolites. 2025 Aug 3;15(8):527. doi: 10.3390/metabo15080527 (PMC12388189; doi:10.3390/metabo15080527)
Supplement: Supplementary file 1 [file metabolites-15-00527-s001.zip › metabolites-3668743 Suppl S2.pdf]

Differences in the profile of aromatic metabolites in the corre-sponding blood serum and cerebrospinal fluid samples of pa-tients with secondary bacterial meningitis

Alisa K. Pautova, Peter A. Meinarovich, Vladislav E. Zakharchenko, Pavel D. Sobolev, Natalia A. Burnakova, Natalia V. Beloborodova

Supplementary S2. Statistical Data Analysis

PCA is a method that can be used for dimensional reduction and data visualization. Fig. S1 a) shows a scatter plot of all metabolomics data in serum and CSF projected to 2-dimensional space. We can see that group I and II points are linearly separable (dotted line in Fig. S1 a). Fig. S1 also consists of loadings plots (b, c) that demonstrate loadings per feature (i.e. weights for every feature to compute values for components). Percentages of variance explained by horizontal and vertical components are equal to 25% and 17%, respectively.

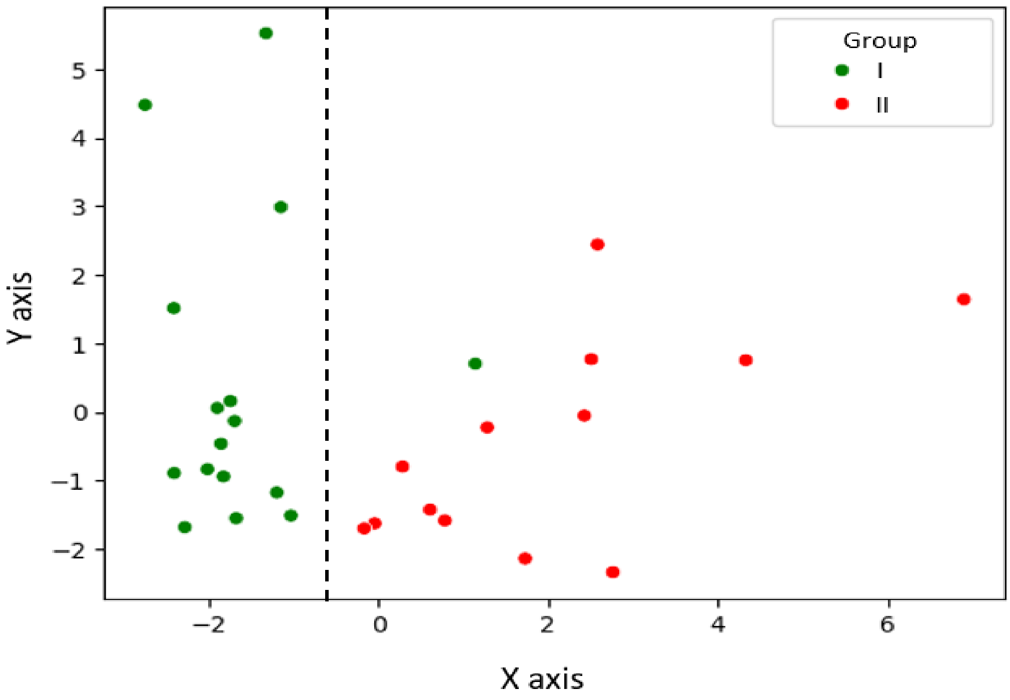

(a)

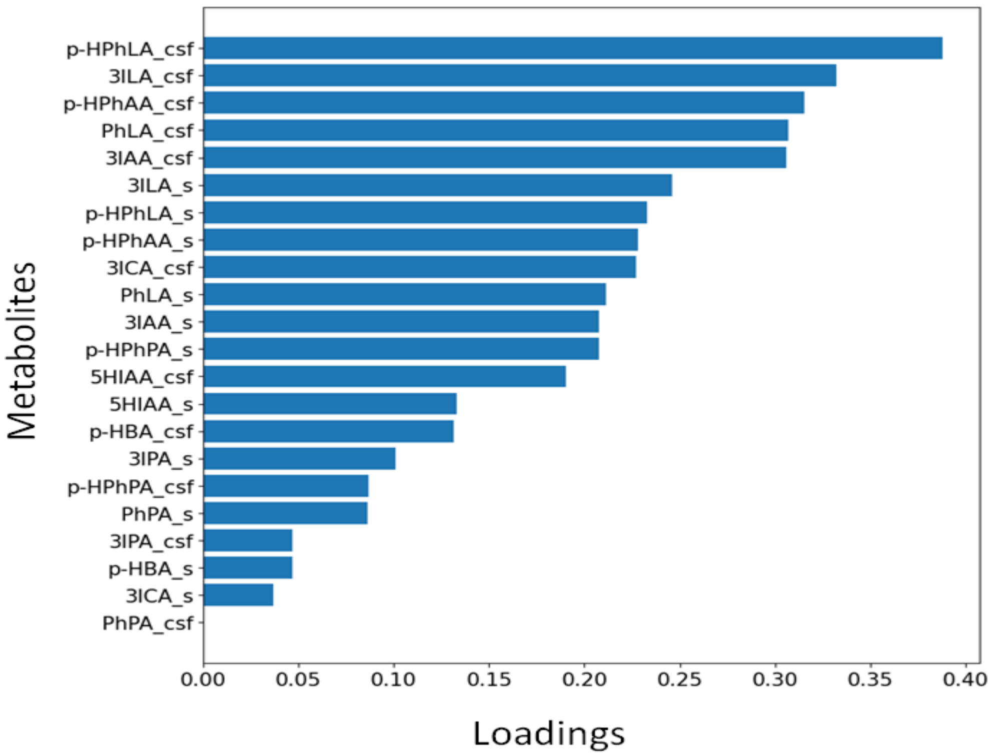

(b)

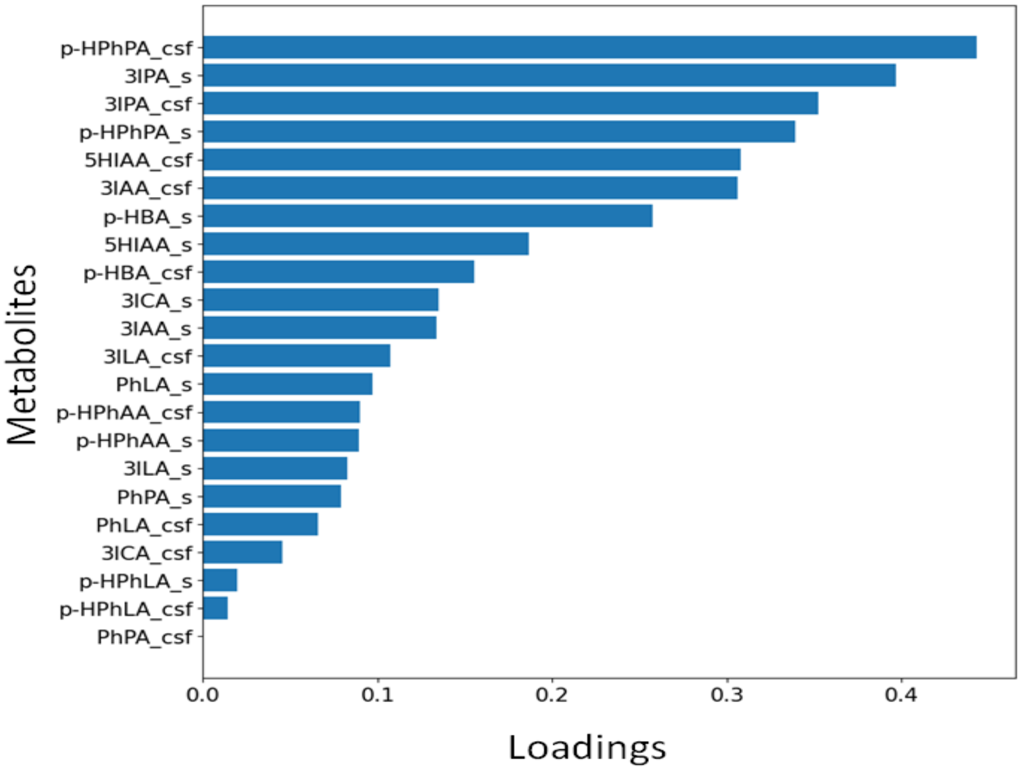

(c)

Figure S1. PCA results for metabolomics data. a) Scatter plot shows that samples from different groups are linearly diverged (dotted line); bar plots show loadings of features for b) horizontal and c) vertical components, respectively.

Evaluation of feature importances was performed with the Boruta algorithm, which compares the importance of every feature with the importance of noisy variables. The finally selected metabolites were p-HPhLA and PhLA in CSF, 3ILA and 3IAA in serum samples (Fig S2 a). The coefficients of this model are shown in Fig. S2 a) and can be considered as the importance of every feature for dividing samples into two groups.

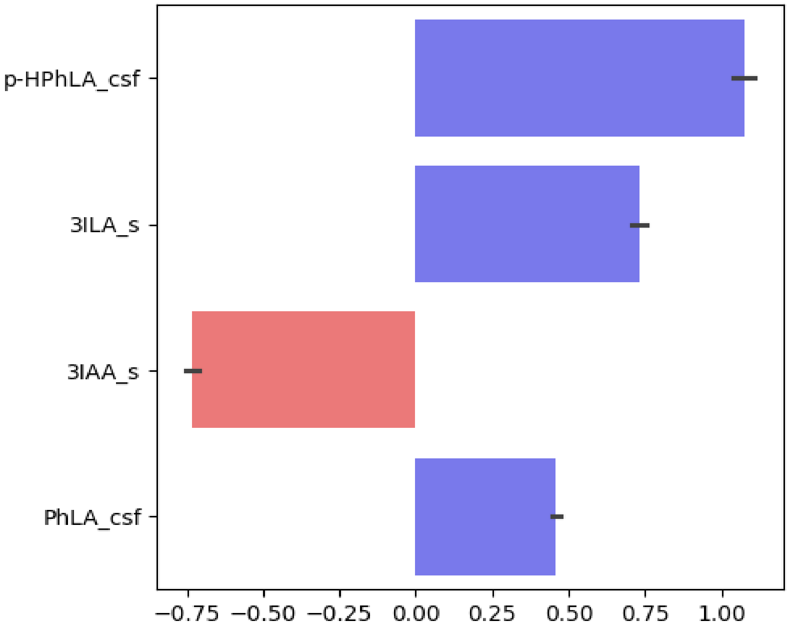

(a)

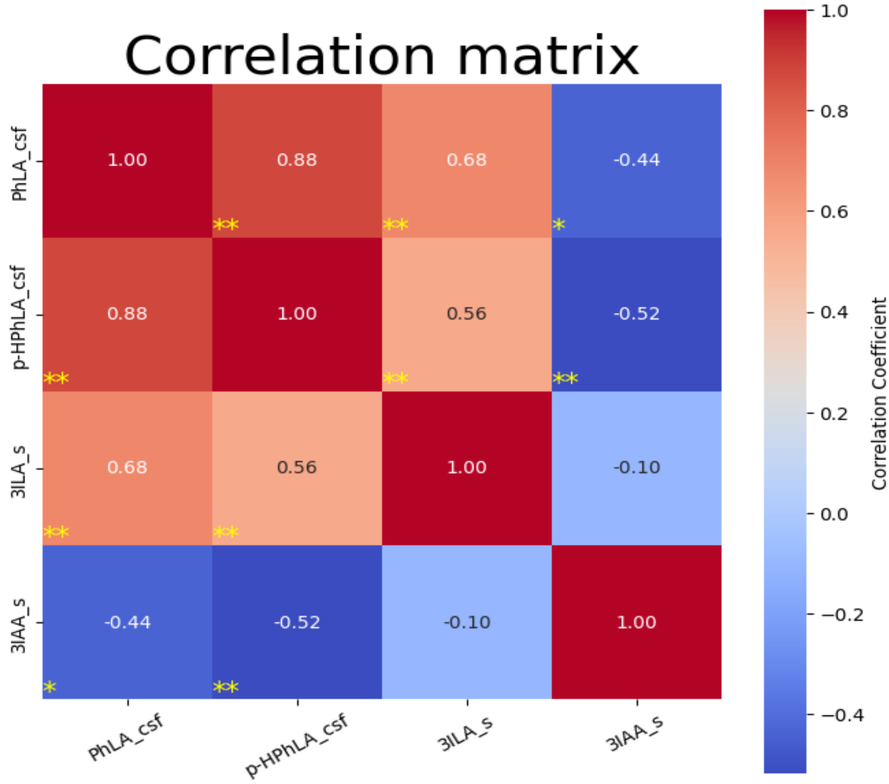

(b)

Figure S2. a) Weights for the SVC model fitted on features, chosen by the Boruta algorithm. Blue bars explain positive values, while red ones respect negative values. 95% CI explained by dark dashes. b) Correlation matrix for selected features on overall dataset (without considering the sample group).

For the features chosen by the Boruta algorithm, we performed ROC analysis to define the opportunity of single metabolites to classify samples into groups. In addition, we computed cut-off values to understand the decision rules of the univariate classifiers. The undisputed leaders were p-HPhLA and PhLA in CSF with cut-off values equal to 1734 and 370 nmol/l, respectively, which accurately classified samples into two groups with 100% sensitivity and specificity. 3ILA in serum also had good prognostic properties with a cut-off value of 829 nmol/l, 92% sensitivity and 89% specificity. The cut-off for 3IAA was 716 nmol/l with 76% sensitivity and 84% specificity (Table S1). We also conduct ROC anal-ysis for CSF leukocytes, neutrophils, and CSF analysis using CDC criteria (presence all of the following criteria: leukocytosis >300 cells/mm3, neutrophils >80%, protein >1 g/l, and glucose <2.7 mmol/l). While leukocytes and neutrophils showed good predictive ability with cut-off values of 147 cells/mm3 and 83%, respectively, united CDC criteria have lower results than previous features (Table S1).

Before the SVC model building, we explored Spearman's correlations between the most important metabolites in all samples without division into groups to reveal if any of metabolites correlate with each other and while using together may put an extra noise in resulting model. PhLA and p-HPhLA in CSF, and PhLA in CSF and 3ILA in serum were highly correlated; hence, there was no reason to include PhLA in CSF in the overall model (Fig. S2 b). After normalization, the resulting SVC model showed excellent classification ability fitted on these three metabolites (Table S1).

Table S1. ROC-AUC, sensitivity (true positive rate, TPR), and specificity (1 - false positive rate, 1-FPR) for univariate and multivariate models to classify samples into groups. For single variables, the cut-off values are demonstrated.

Model/variable name	Cut-off value	ROC-AUC, 95% CI	Sensitivity, 95% CI	Specificity, 95% CI
**Univariate models using most common criteria**				
Neutrophils, %	83	0.94 (0.88, 0.97)	0.84 (0.75, 0.93)	1.00 (1.00, 1.00)
Leucocytes, cells/mm^3	147	0.93 (0.86, 0.97)	0.85 (0.73, 0.93)	1.00 (1.00, 1.00)
Laboratory CDC criteria	-	0.75 (0.67, 0.80)	0.85 (0.72, 0.92)	0.63 (0.52, 0.75)
**Univariate models using aromatic metabolites**				
p-HPhLA in CSF, nmol/l	1734	1.00 (0.99, 1.00)	1.00 (1.00, 1.00)	1.00 (1.00, 1.00)
PhLA in CSF, nmol/l	370	1.00 (0.99, 1.00)	1.00 (1.00, 1.00)	1.00 (1.00, 1.00)
3ILA in serum, nmol/l	829	0.94 (0.91, 0.97)	0.92 (0.77, 0.98)	0.89 (0.82, 1.00)
3IAA in serum, nmol/l	716	0.83 (0.74, 0.90)	0.76 (0.53, 0.87)	0.84 (0.72, 1.00)
**Multivariate models using aromatic metabolites**				
SVC (*p*-HPhLA in CSF, 3IAA and 3ILA in serum)	-	1.00 (1.00, 1.00)	1.00 (1.00, 1.00)	1.00 (1.00, 1.00)
SVC (3IAA and 3ILA in serum)	-	1.00 (1.00, 1.00)	1.00 (1.00, 1.00)	1.00 (1.00, 1.00)

Moreover, to demonstrate that all points from the same patient were within corresponding group I or II, the dynamics of the most statistically significant parameters from Table S1 are illustrated in Fig. S3 for one patient from group I (red lines) and one patient from group II (green lines). It can be noted that for the patient from group II concentration of serum 3ILA in the 3rd point is above the dotted line that

corresponds to the cut-off values of 829 nmol/L. In this point concentration of serum 3ILA should be classified to group II and reflect that this parameter has not excellent specificity (only 89%).

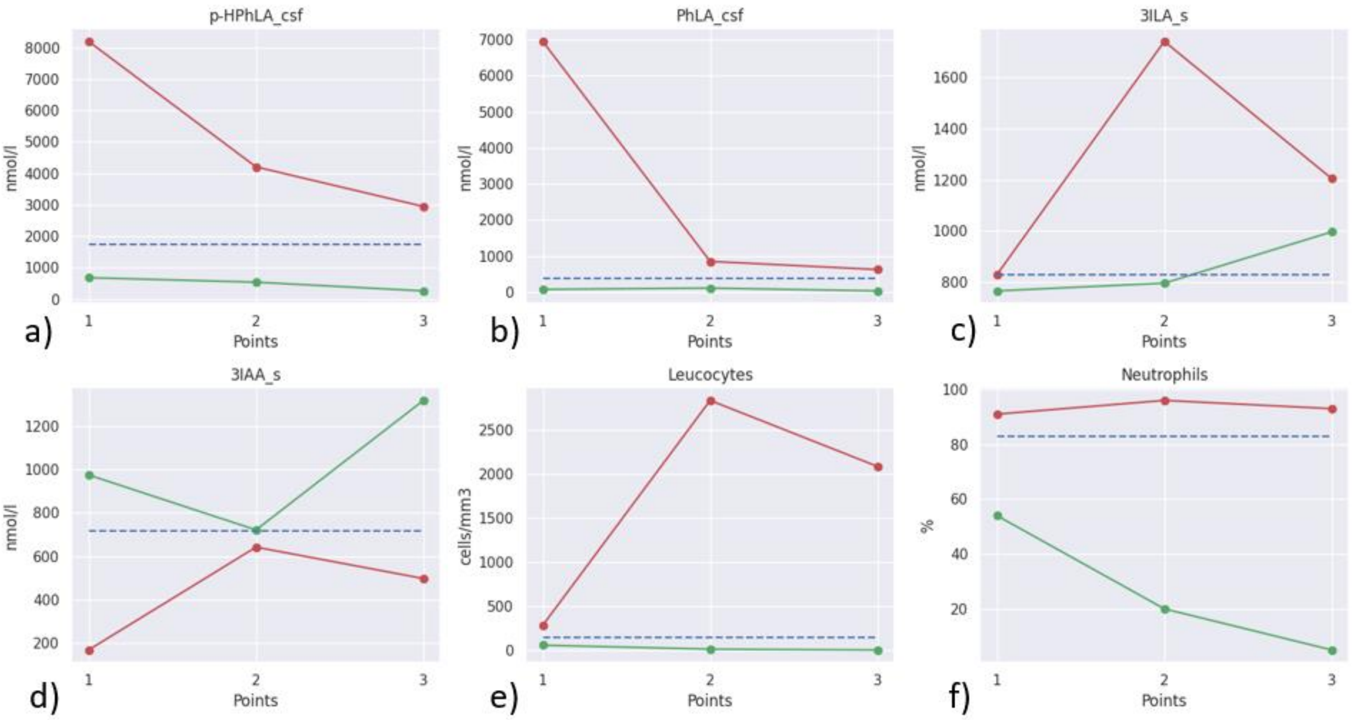

Figure S3. Plots show dynamics of the parameters a) p-HPhLA in CSF (nmol/L), b) PhLA in CSF (nmol/L), c) 3ILA in serum (nmol/L), d) 3IAA in serum (nmol/L), e) leucocytes (cells/mm3), f) neutrophils (%), that were analyzed in Section 3 in three conseqtutive points. Green lines explain a patient from group I and red lines explain a patient from group II. The dotted lines correspond to the cut-off values from Table S1.
